# Safety profile of magnetic sphincter augmentation for gastroesophageal reflux disease

**DOI:** 10.3389/fsurg.2023.1293270

**Published:** 2023-11-07

**Authors:** Caterina Froiio, Alberto Aiolfi, Davide Bona, Luigi Bonavina

**Affiliations:** ^1^ Department of Biomedical Science for Health, Division of General and Foregut Surgery, IRCCS Policlinico San Donato, University of Milan, Milan, Italy; ^2^ Department of Biomedical Science for Health, Division of General Surgery, IRCCS Galeazzi-Sant’Ambrogio, University of Milan, Milan, Italy

**Keywords:** magnetic sphincter augmentation device, LINX, erosion, dysphagia, MSA removal, gastroesophageal reflux disease (GERD)

## Abstract

**Background:**

The magnetic sphincter augmentation (MSA) procedure is an effective treatment for gastroesophageal reflux disease (GERD). Adverse events requiring MSA device removal are rare, but the true prevalence and incidence may be underestimated.

**Methods:**

Retrospective study on a prospectively collected database. Patients who underwent MSA procedure between March 2007 and September 2021 in two tertiary-care referral centers for esophageal surgery were included. The trend of MSA explant, the changes in the sizing technique and crura repair over the years, the technique of explant, and the clinical outcomes of the revisional procedure were reviewed.

**Results:**

Out of 397 consecutive patients, 50 (12.4%) underwent MSA removal, with a median time to explant of 39.5 [IQR = 53.7] months. Main symptoms leading to removal were dysphagia (43.2%), heartburn (25%), and epigastric pain (13.6%). Erosion occurred in 2.5% of patients. Smaller (12- and 13-bead) devices were the ones most frequently explanted. The majority of the explants were performed laparoscopically with endoscopic assistance. There was no perioperative morbidity, and the median length of stay was 2.8 ± 1.4 days. After 2014, changes in sizing technique and crura repair resulted in a decreased incidence of explants from 23% to 5% (*p* < 0.0001). Multivariate analysis confirmed the protective role of added bead units [HR 0.06 (95% CI = 0.001–0.220); *p* < 0.000].

**Conclusion:**

Oversizing and full mediastinal dissection with posterior hiatoplasty may improve the outcomes of the MSA procedure and possibly reduce removal rates.

## Introduction

The magnetic sphincter augmentation (MSA) device, first introduced in 2007 and approved by FDA in 2012, has gained increasing acceptance as a valid therapeutic option for the management of gastroesophageal reflux disease (GERD) (1–3). MSA has been shown to be effective with relief of reflux symptoms, discontinuation of daily proton pump inhibitors (PPI), and reduction of esophageal acid exposure. Compared to the traditional fundoplication, MSA seems associated with less gas bloat symptoms and maintained ability to belch and vomit (4–6). Other potential advantages of MSA are procedural standardization and preservation of esophago-gastric anatomy (7–12).

However, concerns about MSA-related complications, such as persistent dysphagia and full-thickness erosion, have led to criticism mainly based on historical and anecdotal evidence with the Angelchick prosthesis (13). Previous studies reported MSA device removal rates ranging from 1.1% to 6.7% (14–17). While the use of the MSA is increasing worldwide, studies focusing on device failure, explant techniques, and clinical outcomes of revisional surgery remain essential at this stage of MSA adoption in surgical practice. We aimed to analyze the long-term safety profile of MSA and to identify factors predictive for removal.

## Materials and methods

### Study design

A retrospective, observational two-center cohort study was designed and approved by the local Institutional Review Board of two tertiary care University hospitals (IRCCS Policlinico San Donato and IRCCS Ospedale Galeazzi-Sant’Ambrogio). The prospectively collected dataset was queried to identify adult patients who underwent MSA device implant and/or removal from March 2007 and September 2021 with 12-month minimum follow-up. Patients with abnormal esophageal acid exposure and persistent GERD symptoms despite maximal PPI therapy for at least 6 months were eligible for MSA implant. Patients with severe esophageal dysmotility, previous esophageal surgery, known allergy to titanium/nickel, or eating disorders were excluded. Patients were divided into two groups, MSA-R (removed) and MSA-P (preserved), and were compared in terms of demographics, clinical features, procedural characteristics, and postoperative outcomes.

Age, sex, body mass index (BMI), comorbidity, symptoms, duration disease, and duration of PPI use were recorded. Severity of symptoms was measured by means of the Gastroesophageal Reflux Disease Health-Related Quality of Life (GERD-HRQL) scale (18). Preoperative data included upper gastrointestinal endoscopic findings (Hill grade, size of hiatal hernia, Barrett's esophagus, esophagitis), swallow study (hiatal hernia, esophageal dysmotility), conventional or high-resolution esophageal manometry (motility pattern, distal esophageal amplitude, lower esophageal sphincter basal and residual tone, total/intra-abdominal LES length), and 24-hour pH- or pH-impedance monitoring (percent pH <4, total number of reflux episodes).

Collected operative data included year of surgery, MSA device size, number of added bead units after sizing, type of mediastinal dissection (no dissection ND; minimal dissection MD; full dissection FD), and number of stitches required to approximate the crural defect. Operative time, and intra- and postoperative complications graded according by the Clavien-Dindo (C-D) classification were collected (19). Safety of the procedure was defined as no occurrence of major intraoperative or early and long-term complications (C-D ≥3). Patients who underwent explant of the device in other hospitals were interviewed by telephone.

### Surgical technique

The laparoscopic technique for MSA implant evolved over the study period. Since 2014, the original metal sizer ring was replaced by a new sizer, equipped with a soft tip for a dynamic measurement of the esophageal circumference. To determine the proper size of the device to be implanted, care was taken to repeat sizing at least twice and to rotate the shaft at each size for visual inspection of esophageal compression during closure of the tip. A variable number of 1 to 3 beads were added from the point of release of the sizing tool. Since January 2018, full mediastinal dissection was routinely performed in all patients.

**Figure 1:**
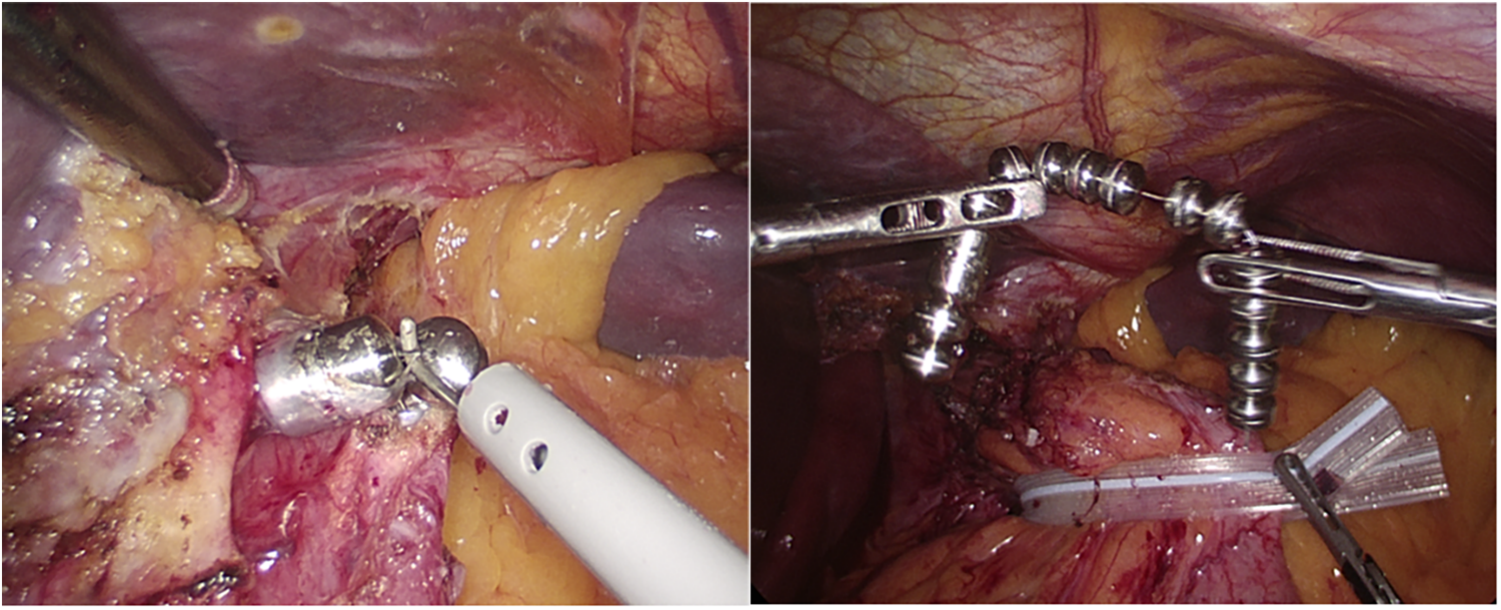
Incision of the fibrotic capsule overlying the MSA device. After cutting the wire connecting the beads, the device is removed and extracted though a 10 mm port.

The surgical technique of laparoscopic MSA explant consisted of the following steps: (1) identification of the fibrous capsule covering the device; (2) incision of the capsule with electrocautery to grasp the anterior beads; (3) unlocking the device or cutting the wire between adjacent beads; (4) progressive retrieval of the entire device from the retroesophageal tunnel; (5) device extraction from the abdominal cavity through a 12 mm port site (Figure 1). Intraoperative endoscopic assistance was used to identify the gastroesophageal junction and to check the integrity of the esophageal mucosa after removal. In some patients, partial or complete retrieval of the MSA device eroded into the esophageal lumen could be performed endoscopically after unlocking the device or cutting the wire connecting the beads. A concurrent cruroplasty with or without partial fundoplication was performed in selected patients. The choice of fundoplication was based on patients’ preference, persistent GERD symptoms, presence of hiatal hernia, and persistently abnormal esophageal acid exposure on 24-hour pH monitoring.

The post-operative follow-up consisted of barium swallow study and/or upper-GI endoscopy within one year after surgery. Clinical evaluation was performed yearly thereafter. All patients completed the GERD-HRQL questionnaire at each follow-up time point. Selected patients underwent esophageal manometry and/or 24-hour pH monitoring.

All procedures were conducted in accordance with the ethical standards and with the Helsinki Declaration. Appropriate informed consent was collected from all patients.

### Statistical analysis

Categoric variables are presented as frequency and percentages while continuous variables are presented as means ± SD for normal distributions or median and interquartile range for non-normal distributions. Event was defined MSA removal. Time to event was definite as the time from surgery to removal. Patients without events at the last follow-up were entered in the survival analysis. The study compared Removal vs. No-Removal patients and within the Removal group, patients with erosion and dysphagia. Median follow-up time was calculated according to the Kaplan–Meier method. The Mann–Whitney *U*-test was used to compare continuous variables, whereas the Fisher's exact test or Pearson's *χ*2 test was used to compare proportions for categorical variables. Multivariate logistic regression analysis was performed to identify independent predictors for MSA removal. Multicollinearity testing was performed to identify the correlation between these variables. The accuracy of the test was calculated using the area under the curve with a 95% confidence interval (CI). Variables with *p* values less than 0.05 were considered significant. All analyses were performed using SAS 9.4 (SAS Institute, Inc, Cary, NC).

## Results

During the study period, 397 patients underwent MSA device implant for refractory GERD. The MSA device explant rate was 12.6%, and the median time from index surgery to device removal was 39.5 [IQR = 53.7] months. Dysphagia (43.2%), heartburn (25%), epigastric pain (13.6%), and erosion (2.5%) were the main reasons for device removal. Two patients were explanted due to the need of 3-T MRI studies. In one patient presenting with de-novo epigastric pain, the chest x-ray showed an unlocked device.

No significant differences were found between patients with and without dysphagia in terms of preoperative symptoms, radiological, endoscopic, manometric findings, and operative technique (type of hiatal dissection, number of crura stitches, and number of added beads). The median time to explant was similar in patients with and without dysphagia (41 vs. 39.5 months; *p* = 0.99). MSA-related erosion occurred in 2.5% of cases, more commonly in patients with smaller (12 and 13 beads) devices. No significant differences were found between patients with and without erosion in terms of preoperative symptoms, radiological/endoscopic/manometric findings, and operative variables.

All patients underwent laparoscopic MSA device removal with endoscopic assistance. There were no conversions to an open procedure. Eight patients consented only for device removal and refused any additional antireflux repair. Two patients underwent a combined single-stage endo-laparoscopic procedure. A 36-month pregnant patient with MSA erosion underwent a two-stage procedure, partial endoscopic removal first and subsequent laparoscopic removal after delivery. A totally endoscopic explant was successfully completed in one patient. Most explanted MSA devices were the 12- and 13-bead. The overall morbidity rate of the revisional procedure was 6% (Tables 1, 2). Toupet fundoplication was performed in patients with recurrent heartburn; in patients that gave consent, an anterior fundoplication was fashioned after MSA explant.

**Table 1:** Operative characteristics and post-operative outcomes of patients undergoing MSA removal.

	*n* = 50
Surgical approach, *n* (%)	
Laparoscopic	46 (92)
Hybrid (Laparo-endoscopic)	3 (6)
Endoscopic	1 (2)
Full crural repair, *n* (%)	
Yes	33 (67.3)
No	16 (32.6)
Antireflux procedure, *n* (%)	
Toupet	22 (44)
Dor	15 (30)
Lortat-Jacob	3 (6.4)
Collis-Nissen	1 (2)
None	9 (17.6)
Operative time, min [median; (IQR)]	117.5 [65]
Length of stay, days (mean ± sd)	2.8 ± 1.4
Grade C–D complications, *n* (%)	
≥3	0 (0)
<3	3 (6)
30–day readmission, *n* (%)	2 (4)
60–day readmission, *n* (%)	0

**Table 2:** Demographic, clinical, and procedural data of the patient population.

	Overall (*n* = 397)	MSA-P (*n* = 347)	MSA-R (*n* = 50)	*p*-value
M/F	264/133	231/113	32/18	0.58
Age, years (mean ± sd)	42.5 ± 13.5	46 ± 13.6	42.1 ± 12.5	0.08
BMI, Kg/m^2^ (mean ± sd)	24.9 ± 3.8	25 ± 3.7	24.6 ± 4.4	0.28
Years of therapy (mean ± sd)	6.7 ± 5.4	6.9 ± 5.5	5.2 ± 4.1	0.06
Reflux symptoms, *n* (%)				0.21
Typical	133 (33.5)	118 (34.1)	15 (29.7)	
Atypical	11 (2.8)	8 (2.3)	3 (6.4)	
Both	253 (63.7)	221 (63.6)	32 (63.9)	
Preoperative GERD-HRQL score Median; [IQR]	20 [9]	6.8 [6.4]	9 [8]	0.9
Preoperative DeMeester score Median; [IQR]	28.9 [25.3]	28.25 [25]	31.5 [25.2]	0.09
Surgical procedures, *n* (%)				0.87
MSA	151 (38.1)	133 (38.4)	18 (36.4)	
MSA + crura repair	246 (61.9)	214 (61.6)	32 (63.6)	
MSA size, *n* (%)				<0.001
11	8 (2)	5 (1.4)	3 (6)	
12	60 (15)	40 (11.6)	21 (42)	
13	68 (17.1)	57 (16.4)	11 (22)	
14	98 (24.7)	89 (25.7)	9 (18)	
15	103 (26)	99 (28.5)	3 (6)	
16	58 (14.6)	54 (15.6)	3 (6)	
17	2 (0.6)	3 (0.8)	0	

MSA, magnetic sphincter augmentation; MSA-P(Preserved); MSA-R(Removed); BMI, body mass index; GERD-HRQL, gastro-esophageal reflux disease health related quality of life.

**Figure 2:**
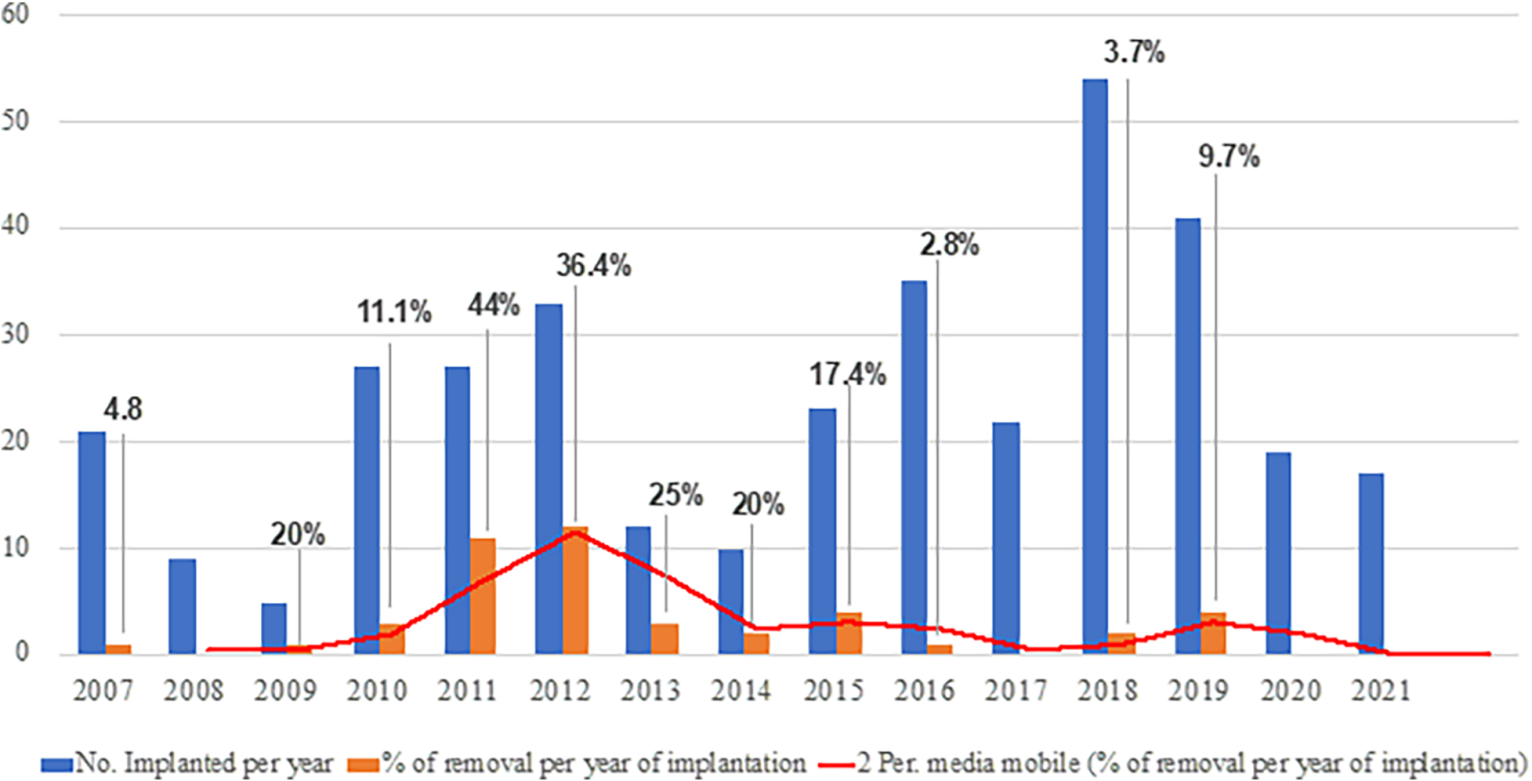
Rate of MSA device removal per year of implantation.

**Figure 3:**
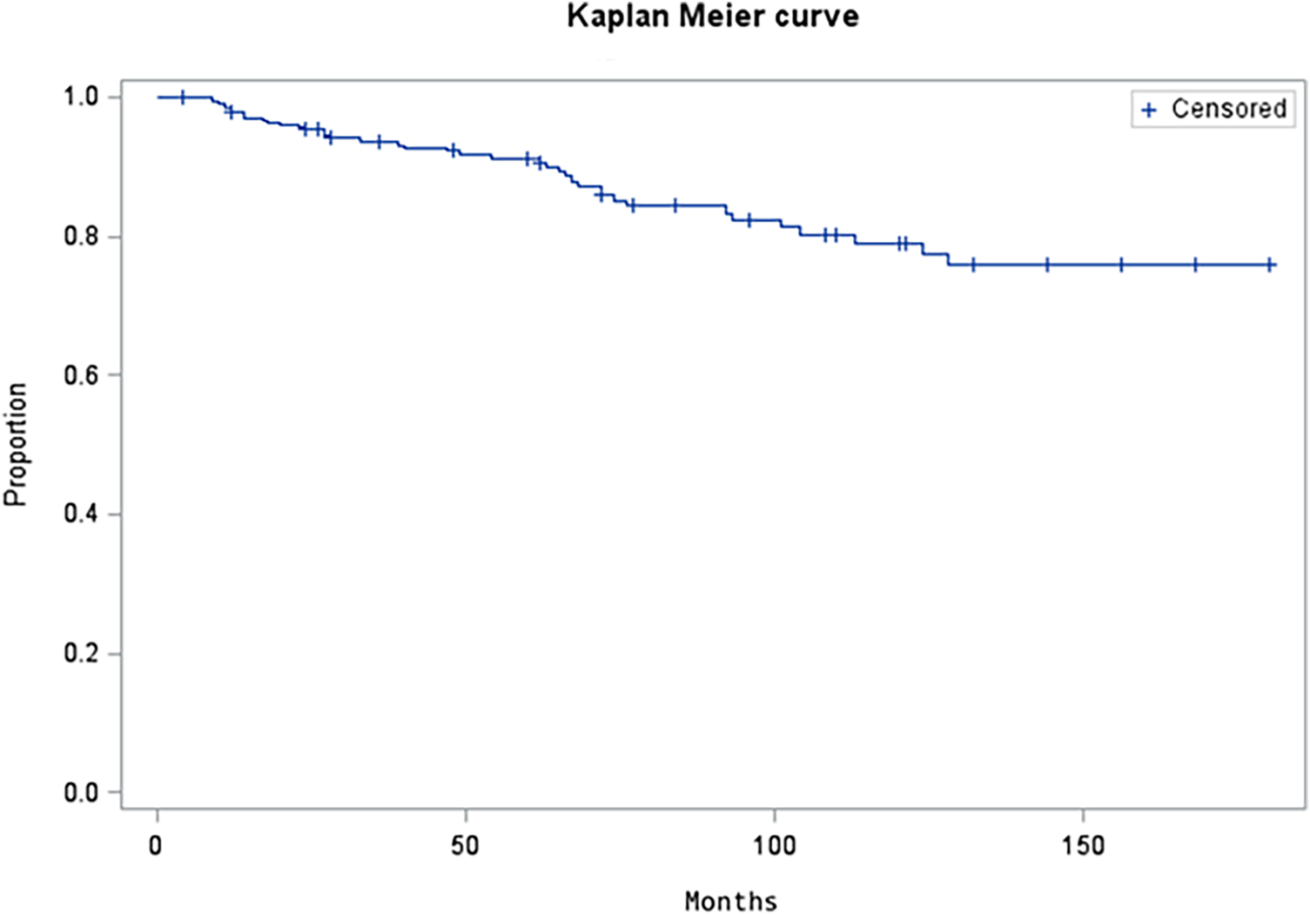
Kaplan meier curve showing the cumulative MSA explant rate.

The highest rates of explants were recorded between the years 2009 and 2014 (Figure 2). Since 2014, upon modification of the surgical procedure, the incidence of explants significantly decreased from 23% to 5% (*p* < 0.0001). The removal rate according to the number of added beads was also significantly lower in patients with at least 2 added units (56.8% vs. 18%; *p* < 0.0001). At the 60-month follow-up, with a total of 196 patients observed, the cumulative risk of explant was 9.9%. The Kaplan Meier curve shows the trend of device removal after MSA implantation (Figure 3).

Preoperative hiatus hernia, greater percent of acid exposure in the supine position, no or minimal hiatal dissection, use of non-iterative sizer ring, and use of smaller devices were significantly more prevalent in the removal group at univariate analysis (Table 3). Hiatal hernia at the time of diagnosis (OR 7.03, 95% CI = 2.93–16.89), the number of added units (OR 3.25, 95% CI = 1.36–7.80), and the type of hiatal dissection (OR 2.65 (95% CI = 1.38–5.10) were risk factors for MSA explant. Use of a larger device was associated with a reduced risk for removal (OR 0.30, 95% CI = 0.12–0.72). In the logistic regression analysis, hiatal hernia at time of diagnosis was an independent predictor of MSA device explant (OR 18.1, 95% CI = 8.80–37.26; *p* < 0.0001), while oversizing with added bead units appeared to be a protective factor (OR 0.06, 95% CI = 0.001–0.22; *p* < 0.0001) (Table 4).

**Table 3:** Comparison between the MSA-preserved and MSA-removed group.

	MSA-P (*n* = 347)	MSA-R (*n* = 50)	*p*-value
Preoperative findings			
Pre-operative diagnosis, *n (%)*			0.01
GERD	198 (57.1)	16 (36)	
GERD + HH	149 (42.9)	32 (64)	
Hiatal hernia, length (cm) (mean ± sd)	1.76 ± 1.4	1.61 ± 1.17	0.57
pH-metric features			
% Total time pH <4	6.80 (6.4)	8.00 (5.90)	0.09
% pH <4 upright	7.50 (8.0)	3,00 (9.25)	0.72
%pH <4 supine	3.8 (9.0)	7.2 (9.75)	0.02
Manometric features			
LES resting pressure (mmHg)	15.10 (14)	15.00 (16)	0.8
LES residual pressure (mmHg)	6.30 (7.6)	9.8 (2.8)	0.05
LES length (cm)	2.25 (2.4)	3.00 (1.0)	0.13
LES intrabdominal length (cm)	1.00 (2.0)	0.65 (2.3)	0.87
DEA (mmHg)	66.50 (36.3)	59.00 (35.0)	0.27
% peristalsis	100.00 (20.0)	100.00 (10.0)	0.43
Index procedural data			0.87
Surgical procedure, *n (%)*			
MSA	133 (38.3)	18 (36)	
MSA + Crura repair	214 (61.7)	32 (64)	
Crura repair stitches, *n (%)*			0.08
0–2	232 (66.8)	42 (84)	
3–5	115 (33.2)	8 (16)	
Hiatal dissection, *n (%)*			0.001
ND	136 (39.2)	18 (36)	
MD	78 (22.5)	26 (52)	
FD	133 (38.3)	6 (12)	
Sizing tool, *n (%)*			<0.0001
Metal ring	113 (32.5)	35 (70)	
New size	234 (67.5)	15 (30)	
Added bead units, *n (%)*	206 (59.4)	9 (18.2)	<0.0001
No. added bead units, *n (%)*			<0.0001
0–1	150 (43.2)	41 (82)	
2–3	197 (56.8)	9 (18)	
Operative time, min mean *(ds)*	63.5 ± 29.3	51.2 ± 18.9	0.002
Year of surgery			<0.0001
<2014	118 (34)	37 (74)	
>2014	229 (66)	13 (26)	

GERD, gastroesophageal reflux diseas; HH, hiatus hernia; LES, lower esophageal sphincter; DEA, distal esophageal amplitude; MSA, magnetic sphincter augmentation; ND, no dissection; MD, minimal dissection; FD, full dissection.

**Table 4:** Univariate and multivariate analysis analyzing outcomes of MSA removal.

	Univariate		Multivariate	
	HR	*p*-value	HR	*p*-value
% pH <4 supine position	1.02 (0.99–1.05)	0.14	–	–
GERD + HH vs. GERD	7.03 (2.93–16.89)	<0.0001	18.11 (8.80–37.26)	<0.0001
New Sizer tool vs. Metal ring	0.47 (0.23–0.97)	0.04	–	–
Hiatal dissection		0.003	–	0.91
FD	0.89 (0.32–2.48)	–	0.97 (0.37–3.25)	–
MD	2.65 (1.38–5.10)	–	0.88 (0.48–1.60)	–
ND	Ref	–	Ref	–
Added unit (yes vs. no)	0.30 (0.12–0.72)	0.005	0.06 (0.01–0.22)	<0.0001
No. added unit (0–1 vs. 2–3)	3.25 (1.36–7.80)	0.008	–	–
Years of surgery (>2014 vs. ≤2013)	0.42 (0.20–0.88)	0.03	1.29 (0.32–5.17)	0.72

GERD, gastroesophageal reflux disease; HH, hiatus hernia; ND no dissection; MD, minimal dissection; FD, full dissection.

## Discussion

In our study, the rate of MSA removal was 12.6%, with a median time to explant of 39.5 months. The main reasons leading to device removal were dysphagia and persistent heartburn/epigastric pain. Device oversizing and full hiatoplasty improved the long-term success rate of the MSA procedure.

Removal of the MSA device is reported in 1.1% to 6.7% of the patients (14–17), with a median time to explant ranging from 3 to 24 months. Reasons for the higher removal rate in our series are twofold. First, previous published studies (9–11) are based on FDA centralized data and self- reporting MAUDE database, therefore complications may be underreported. Second, our study has a long median follow-up and includes a large number of patients implanted during the early years of experience. The main reasons for MSA removal reported in previous series were dysphagia and persistent GERD symptoms (15, 16). Although MSA-related dysphagia was thoroughly investigated in our study, we were unable to find predictors of postoperative dysphagia. Despite the lack of statistical significance, post-operative dysphagia requiring MSA removal occurred more frequently with smaller device sizes. This is similar to a recent retrospective series of 268 patients with a median follow-up of 23 months showing that MSA device size <13 was the only factor associated with postoperative dysphagia (20). Ayazi et al. (21), in a cohort study of 380 patients, found that risk factors for postoperative dysphagia were preoperative dysphagia and less than 80% peristaltic contractions on preoperative manometry. Another recent paper showed that preoperative dysphagia was the only factor significantly associated with postoperative dysphagia (22). Since dysphagia remains the most frequent side effect of MSA placement, it remains of paramount importance to establish the proper post-operative management to prevent the formation of a tight fibrous capsule (23, 24). In particular, early swallowing exercise with frequent small and solid meals appears to be crucial after MSA implant. Ayazi et al. (21) found persistent postoperative dysphagia in 15.5% of the patients, one third of whom required at least one endoscopic dilatation. It appears that early dilation (<8 weeks) worsened dysphagia, probably due to an increased inflammatory response around the device, so the authors advised steroid pulses and delayed endoscopic dilation. In our cohort, the dilation rate was 5.2%, lower than that reported in literature, and the median time to postoperative dilation was longer (13 months after MSA placement).

Mucosal erosion and device migration is the most feared complication and a relevant parameter of safety after any foreign body placement at the EGJ. The overall prevalence in our series was 2.5%, with median implant duration of 59 months. The erosion occurred with device size 12 in most cases, and this is consistent with the analysis of the manufacture database (11). The association of dysphagia/erosion with a smaller device can reasonably be explained by malfunctioning of the device due to a constrictive capsule fibrosis as a result of an exaggerated inflammatory response. All the erosion events reported in literature, as well as in our experience, were non-acute on presentation and were not associated with intrabdominal abscess or peritonitis.

We also assessed the safety of the MSA explant. All procedures were performed electively, by a single stage laparoscopic or combined endo-laparoscopic approach; when migration of beads was found, partial transoral removal of the device was occasionally performed without complications (16, 25). In a retrospective study of 425 patients with a removal rate of 5.5%, Tatum et al. (15) suggested to avoid additional intervention and to redo an MSA implant or perform fundoplication only in patient with malposition of the device or hiatal hernia. In a recent study, removal for dysphagia yielded excellent outcomes regardless of concurrent antireflux repair, whereas patients with persistent GERD had worse outcomes without antireflux repair ( 26). Our preference is to perform a posterior Toupet fundoplication in patients with hiatal hernia or persistent GERD, and an anterior fundoplication in patients with erosion.

The size of the MSA device appears critical to avoid esophageal constriction and to reduce an excessive capsular fibrosis around the device. In our series, from 2007 to 2013, measurement of esophageal circumference was performed using a metal sizing tool (1), and smaller devices were used, which are no longer available. Since 2014, the new standard was to repeat sizing at least twice, rely on visual cues, and oversize by 1 to 3 beads from the point of release of the sizing tool to reduce the risk of mismatch between the device and the esophageal lumen size. These modifications led to the use of larger devices and decrease in the rate of both dysphagia and erosions. More recently, a novel technology of intraoperative impedance planimetry assessed by Functional Lumen Imaging Probe (FLIP) was used for real-time evaluation of esophago-gastric junction distensibility to calibrate the surgical procedure and optimize outcomes. However, more data and normative FLIP values are needed to define the role of this technology in selecting the appropriate device size (27, 28).

In our study, on multivariate analysis, the addition of beads resulted to be an independent protective factor, while the presence of hiatal hernia with GERD was associated with an increased risk of device removal. Interestingly, a few retrospective studies demonstrated favorable short- and medium-term results for patients with >3 cm hiatal hernia (29–32). Irribarra et al. (33) and Tatum et al. (34) found that concurrent hiatal dissection with restoration of esophageal length and crura repair during the MSA procedure were more likely to normalize postoperative DeMeester score and acid exposure, reduce the rate of recurrence/progression of hiatal hernia, and reduce revisional surgery rates compared to minimal hiatal dissection. According to the hypothesis of the sphincter-like action of the diaphragmatic crura (35), the concept of full hiatoplasty was progressively incorporated into the MSA procedure. Inadequate mediastinal dissection and suboptimal crura repair may explain some failures of the MSA procedure. In fact, comparison of the subgroup of patients with hiatal hernia in our series seems to corroborate this analysis. Smaller hernia size (<3 cm) and minimal dissection were associated to worse outcomes. Therefore, patients with larger hernias are likely to have received adequate mediastinal dissection, compared to patients with smaller hernias in whom a less invasive hiatal repair was chosen. These results are not confirmed in our multivariate analysis model probably due to the small sample size, the low rate of removal events, and the retrospective study design.

This study has several limitations. Its retrospective design does not allow for patient stratification based on pre-established clinical and preoperative characteristics. Patients’ selection criteria for MSA changed during the study period, with the extension of the indications to patients who would have previously been considered ineligible for MSA. On the other hand, preoperative assessment has also evolved during the study period. Finally, the relatively small sample size of explanted patients did not allow to identify additional predictive factors with adequate statistical power to reach robust conclusions.

## Conclusion

Technical advances in the MSA procedure, including formal mediastinal dissection, posterior crura repair, and use of a larger device, have improved clinical outcomes and reduced removal rates over the years. Explant of the device performed in a tertiary center by experienced surgeons has proven safe and effective through either a laparoscopic or endoscopic approach. Nevertheless, more data are needed to improve patient selection, long-term safety, and outcomes of the MSA procedure.

## Data Availability

The original contributions presented in the study are included in the article/Supplementary Material, further inquiries can be directed to the corresponding author.
